# *ThASR3* confers salt and osmotic stress tolerances in transgenic *Tamarix* and *Arabidopsis*

**DOI:** 10.1186/s12870-022-03942-w

**Published:** 2022-12-14

**Authors:** Yu Zhang, Huijun Ma, Tianchang Zhou, Zhenyu Zhu, Yue Zhang, Xin Zhao, Chao Wang

**Affiliations:** 1 State Key Laboratory of Tree Genetics and Breeding, Northeast Forestry University, 26 Hexing Road, 150040 Harbin, China; 2 State Key Laboratory of Tree Genetics and Breeding, Key Laboratory of Tree Breeding and Cultivation of the State Forestry Administration, Research Institute of Forestry, Chinese Academy of Forestry, Beijing, 100091 China

**Keywords:** Abiotic stress, ASR protein, Gene expression, ROS-scavenging capability, *Tamarix hispida*

## Abstract

**Background:**

*ASR* (abscisic acid-, stress-, and ripening-induced) gene family plays a crucial role in responding to abiotic stresses in plants. However, the roles of *ASR* genes protecting plants against high salt and drought stresses remain unknown in *Tamarix hispida*.

**Results:**

In this study, a salt and drought-induced *ASR* gene, *ThASR3*, was isolated from *Tamarix hispida*. Transgenic *Arabidopsis* overexpressing *ThASR3* exhibited stimulating root growth and increasing fresh weight compared with wild-type (WT) plants under both salt and water deficit stresses. To further analyze the gain- and loss-of-function of *ThASR3*, the transgenic *T. hispida* plants overexpressing or RNA interference (RNAi)-silencing *ThASR3* were generated using transient transformation. The overexpression of *ThASR3* in *Tamarix* and *Arabidopsis* plants displayed enhanced reactive oxygen species (ROS) scavenging capability under high salt and osmotic stress conditions, including increasing the activities of antioxidant enzymes and the contents of proline and betaine, and reducing malondialdehyde (MDA) content and electrolyte leakage rates.

**Conclusion:**

Our results indicate that *ThASR3* functions as a positive regulator in *Tamarix* responses to salt and osmotic stresses and confers multiple abiotic stress tolerances in transgenic plants, which may have an important application value in the genetic improvement of forest tree resistance.

**Supplementary Information:**

The online version contains supplementary material available at 10.1186/s12870-022-03942-w.

## Background

Different kinds of abiotic stresses are able to alter various traits, genes expression and proteomic profile of different plants [1–4]. Plants sessile in soil consistently encounter abiotic stresses that often limit plant growth and production [5–7]. Plants have evolved different adaptation mechanisms to deal with various abiotic stresses, for example, transcriptional regulation of transcript abundance [8]. Numerous genes, such as molecular chaperones (Hsp60 and Hsp70) and transcription factor genes (*MYBs*, *bZFPs*, *NACs* etc.), play pivotal roles in regulating various signaling pathways and biological processes in response to abiotic stresses [9–11].

ASR proteins are highly hydrophilic with low molecular weight that belong to plant tissue-specific DNA-binding proteins [12]. ASR family members usually harbor a highly conserved abscisic acid/water-deficit stress (ABA/WDS) domain (Pfam PF02496 [13]. *ASR1*, the first reported member of the ASR family, was isolated from the tomato fruit under water-stress conditions [14]. Subsequently, many *ASR* homologs have been discovered from dicot and monocot plants, such as tomato, maize, and wheat [15–17]. However, *ASR* homologous genes are not present in the model plant *Arabidopsis* [12]. ASR proteins were firstly detected solely in the nucleus, whereas they were subsequently found in both the nucleus and the cytosol [7, 18]. It is speculated that ASRs have dual molecular functions in plant cells. Some ASR proteins were considered as transcription factors that bind to specific DNA in a Zn^2+^-dependent manner to regulate the expression of downstream genes, while a few ASR proteins might also act as molecular chaperones to protect the activities of cellular components when present in the cytosol [19–21].

Previous studies have proved that ASR proteins participate in plant growth, senescence, and fruit ripening [12, 22–24]. Moreover, increasing evidence has demonstrated the critical roles of ASR family members in responses to various abiotic stresses [25–27]. The *ASR* gene from banana (*MpASR*) and lily (*LLA23*) conferred drought tolerance in transgenic *Arabidopsis* [24, 28]. *OsASR5*, which contains an HSP and 2OG-Fe (II) oxygenase protein, was speculated to function as a chaperone to enhance drought tolerance in *Arabidopsis* and rice [20]. Similarly, overexpression of *SiASR1* from *Foxtail millet* or *TaASR1* from wheat enhances high salt and drought tolerance in transgenic tobacco via increasing expression levels of reactive oxygen species (ROS)-related genes and activating the antioxidant system [17, 29]. Moreover, ASR1 protein from rice confers stress resistance of yeast cells by scavenging ROS via converting H_2_O_2_ to H_2_O and performing chaperone-like activities [30]. Overexpression of *PheASR2* from *Moso bamboo* in rice showed a high expression of ROS-scavenging related genes [31]. Nevertheless, despite widespread reports of *ASR* genes responding to abiotic stresses, the underlying molecular processes and physiological relevance of *ASR* genes to abiotic stress tolerance remain unclear in *Tamarix hispida*.

*Tamarix hispida* grows as shrubs or tiny trees that are resistant to salt, drought, and harsh temperatures, suggesting the valuable role of this plant species in the functional characterization of stress tolerance-related genes, as well as stress tolerance mechanisms [32, 33].

In this study, an *ASR* gene (*ThASR3*) was cloned from *Tamarix hispida*, which was strongly induced by salt and drought stresses. The role of *ThASR3* in salt and osmotic stress tolerances was demonstrated and elucidated the physiological regulation mechanism of this gene under stress. This study provides candidate gene resource for molecular breeding to improve plant stress tolerance.

## Results

### Gene isolation and sequence analysis of *ThASR3*

**Fig. 1:**
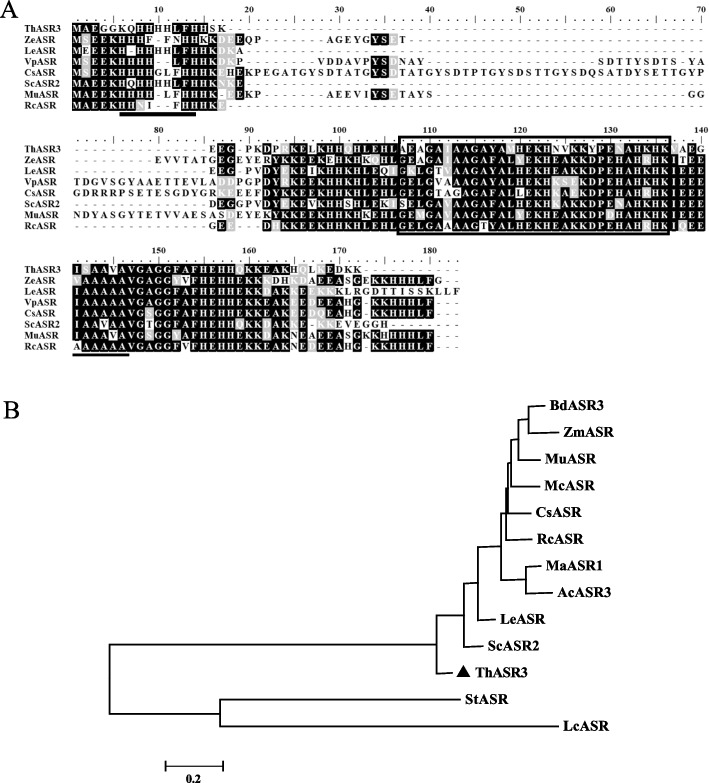
Phylogenetic analysis and multiple alignments of ThASR3 protein. **A** Multiple alignments of ThASR3 protein sequence with those of other seven plants ASRs. BioEdit software was utilized to align amino acid sequences. **B** The phylogenetic tree of ThASR3 and other ASR proteins was constructed by the neighbour-joining method. The sequences of the ASR proteins were obtained from the NCBI website (https://www.ncbi.nlm.nih.gov/protein/), and their GenBank accession numbers are shown below. *Tamarix hispida* ThASR3 (OL310472); *Zea mays* ZmASR (EU960308.1); *Vitis pseudoreticulata* VpASR (DQ336286.1); *Brachypodium distachyon* BdASR3 (XP_003577811.1); *Musa AAB Group* MuASR (ACZ60132.1); *Citrus sinensisSc* CsASR1 (NM_001289141.1); *Ricinus communis* RcASR (XM_002524251.2); *Ananas comosus* AcASR3 (OAY74041.1); *Lycopersicon esculentum Mill* LeASR (L08255.1); *Solanum chilense* ScASR1 (CBY05857.1); *Vitis vinifera* VvASR (AAK69513.1); *Solanum tuberosum* StASR (JX839758.1); *Litchi chinensis* LcASR (HQ831448.1); *Brachypodium distachyon* BdASR (XP_003577811.1); *Mesembryanthemum crystallinum* McASR (AAC14177.1); *Musa acuminata subsp. Malaccensis* MaASR1 (XP_009406127.1). Note: Thick box: enzyme ABA/WDS domain; thick line: histidine-rich area; thin line: alanine-rich area

A salt-induced *ASR* gene, *ThASR3* (Genbank accession number: OL310472), was screened from *T.hispida* by RNA-seq with NaHCO_3_ treatment [34]. The open reading frame (ORF) of *ThASR3* is 309 bp in length, encoding 103 amino acids. Blastx analysis revealed that ThASR3 had 70% sequence identity with ASR from *Citrus Sinensis* (Fig. 1A). And ThASR3 exhibited a highly conserved ABA/WDS motif, a histidine-rich region, and two alanine-rich regions. The phylogenetic tree showed that ThASR3 is closely related to the ASRs subfamily (Fig. 1B). To sum up, ThASR3 belongs to ASR subfamily in *T. hispida*.

### *ThASR3* is induced by abiotic stress

**Fig. 2:**
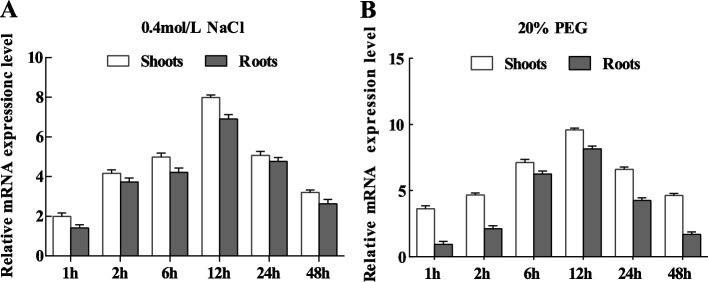
Expression analysis of *ThASR3* in response to salt and drought stresses. The relative mRNA expression level of *ThASR3* in one-month-old *T. hispida* seedlings was detected under 400 mM NaCl **A** and 20% PEG6000 **B** treatments. The relative mRNA expression level of *ThASR3* under mock treatment was designed as 1 to normalize the expression of *ThASR3* under salt or PEG6000 stresses

The expression pattern of *ThASR3* was analyzed in the shoots and roots of *T. hispida* under 400 mM NaCl and 20% PEG6000 treatments. The relative mRNA expression level of *ThASR3* was strongly induced by NaCl or PEG6000 during 1 ~ 12 h and reach the highest level at 12 h in both shoots and roots of *T. hispida* (Fig. 2A, B). These results suggest that *ThASR3* may be involved in the responses to salt and osmotic stresses in *T. hispida*.

### Ectopic expression of *ThASR3* in *Arabidopsis* increases tolerance to salt and osmotic stresses

**Fig. 3:**
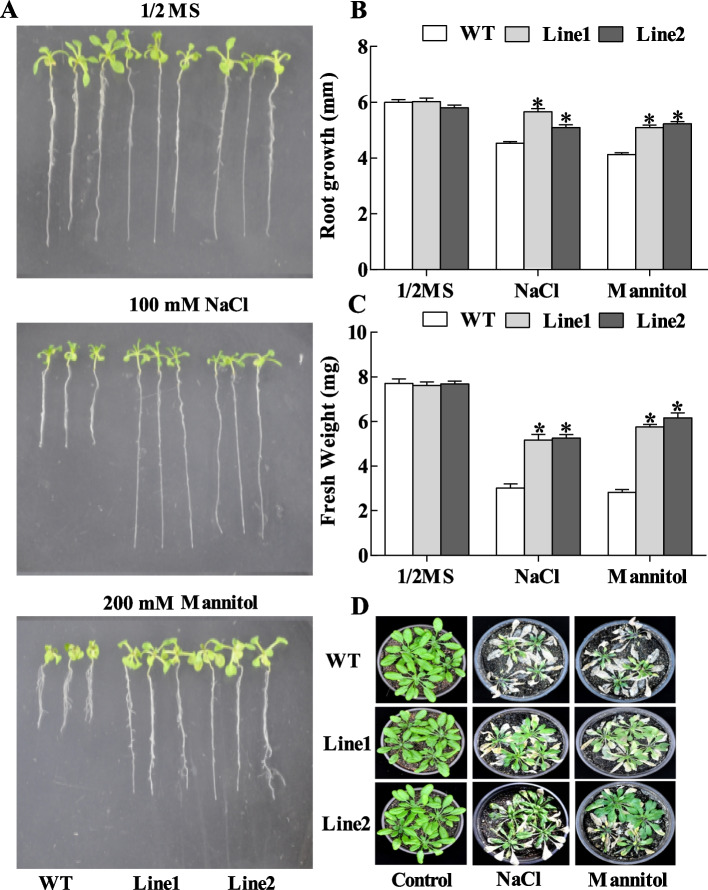
Stress tolerance of overexpressing *ThASR3* transgenic and WT *Arabidopsis* plants. **A** The growth phenotype of *ThASR3* transgenic lines and WT plants. Primary root length **B** and **C** fresh weight analysis under salt (NaCl), osmotic stress (Mannitol) or normal conditions. **D** The phenotypes of the 4-week-old seedlings were photographed after 200 mM NaCl or 300 mM mannitol treatment. Asterisks indicate significant difference compared with control plants (* *P* < 0.05)

To further confirm the function of *ThASR3* in response to abiotic stress, seven independent T3 transgenic *Arabidopsis* lines that ectopic overexpress *ThASR3* were generated. The transcript levels of each line was analyzed using DNA PCR and qRT-PCR. Two *Arabidopsis* transgenic lines with high expression, named Line 1 and Line 2, were selected for subsequent study (Supplementary Fig. S2). Under normal condition, there was no phenotypic difference in fresh weight and root length between transgenic *Arabidopsis* and WT plants. However, when exposed to high salt and mannitol stresses, fresh weight and root length of the transgenic lines were significantly higher than those of WT (Fig. 3A-C). Moreover, overexpression of *ThASR3* apparently enhanced the vegetative growth of transgenic *Arabidopsis* under salt and mannitol stress conditions, compared with WT plants, while no difference was observed under normal conditions (Fig. 3D). Our findings suggest that *ThASR3* plays a positive role in resistance to salt and osmotic stresses in *Arabidopsis*.

### Generation of *T. hispida* plants with transient overexpression or RNAi-silence of *ThASR3*

**Fig. 4:**
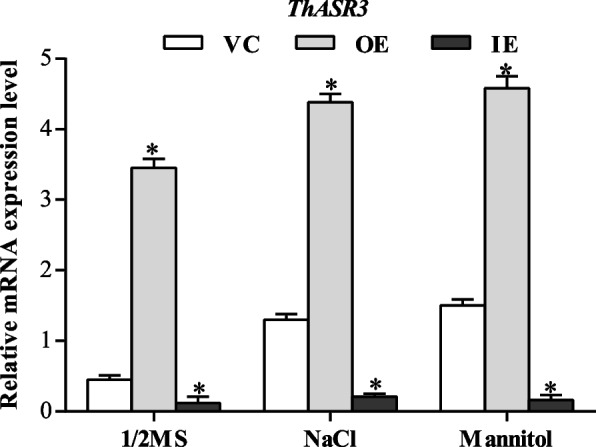
The relative mRNA expression level of *ThASR3* in VC, OE and IE *T. hispida* plantlets. The plantlets were cultivated for 24 h in the normal conditions (1/2 MS medium) or 1/2 MS supplemented with 100 mM NaCl and 200 mM mannitol, and the expression level of *ThASR3* was measured. The asterisks (*) indicate a significant difference (*P* < 0.05) between transformed (OE and IE) and control (VC) plants

To explore the gain- and loss-of-function of *ThASR3*, *ThASR3*-overexpressing *T. hispida* (OE), *ThASR3* RNAi-silenced (IE) plants, and control (empty pROKII vector transformed, VC) plants were generated using a transient expression system, which has been widely used in functional characterization of gene in many plant species [35]. To ensure the accuracy of this experiment, a minimum of three biological replicates, which contain at least 20 transformed *T. hispida* seedlings in each replicate, were performed. The expression level of *ThASR3* in VC, OE, and IE *T. hispida* plants was determined by qRT-PCR, showing that *ThASR3* transcript levels were significantly higher in OE plants and lower in IE plants than VC plants (Fig. 4). The results indicate *ThASR3* can be successfully transformed in *T. hispida* plants by the transient system.

### *ThASR3* improves ROS-scavenging capability

**Fig. 5:**
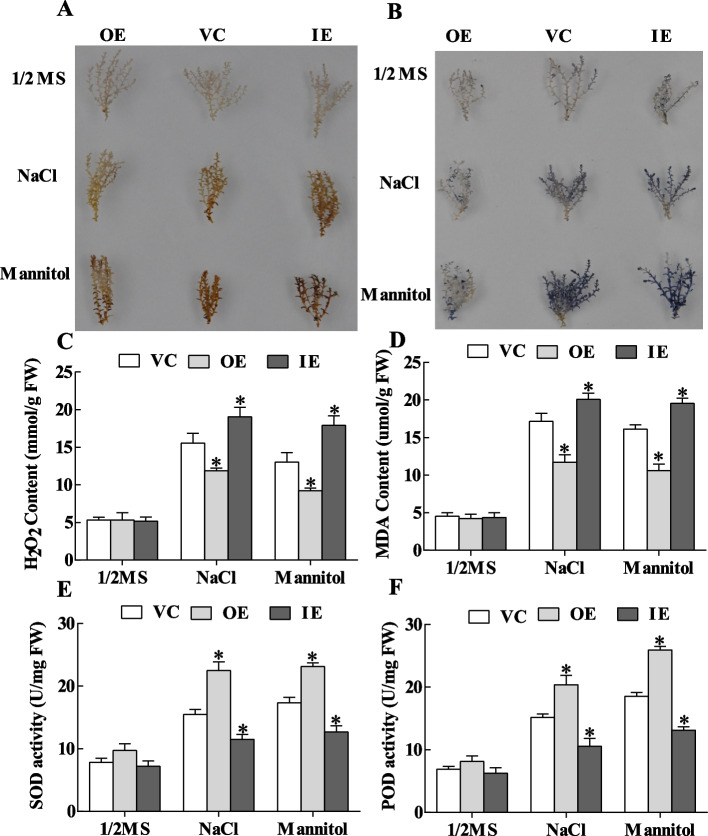
Analysis of ROS accumulation and antioxidant enzyme activities in three types (VC, OE and IE) transgenic *T. hispida* plants under NaCl or mannitol treatment. **A** 3,3′-Diaminobenzidine (DAB) and **B** Nitroblue tetrazolium (NBT) staining were performed to detect H_2_O_2_ and O_2_^·−^ accumulation in young branches of *T. hispida* plants. Analysis of H_2_O_2_ **C** and MDA **D** contents, SOD **E** and POD **F** activities in three types (VC, OE and IE) transgenic *T. hispida* plantlets. 15 plants were selected for photography in each treatment, and the most representative photos were selected and combined. The asterisks (*) indicate a significant difference (*P* < 0.05) between transformed (OE and IE) and control (VC) plants

**Fig. 6:**
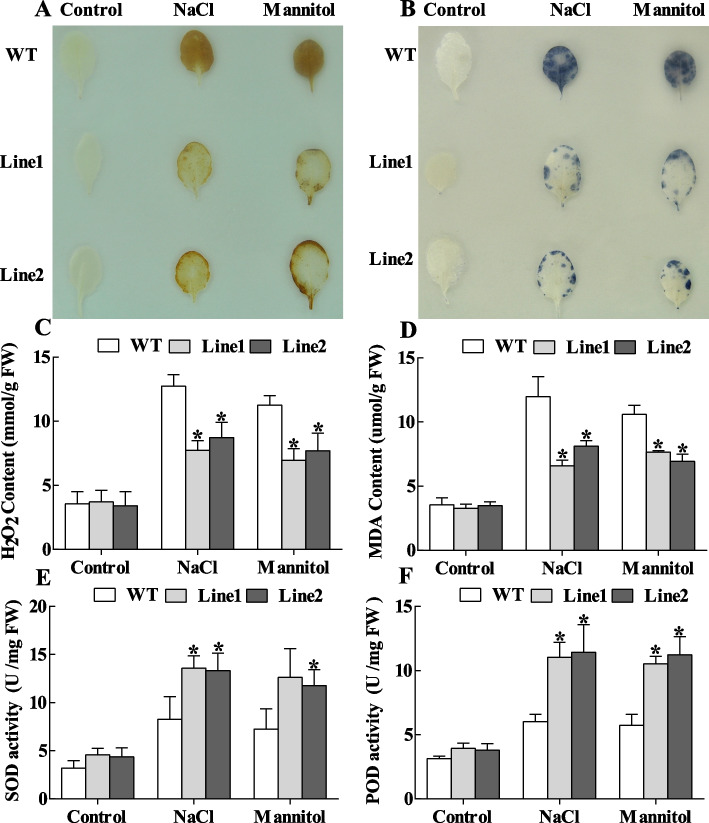
Analysis of ROS accumulation and antioxidant enzyme activities in *ThASR3-*overexpressing *Arabidopsis* and WT plants under NaCl or mannitol treatment. **A** 3,3′-Diaminobenzidine and (DAB) and **B** Nitroblue tetrazolium (NBT) staining were performed to detect H_2_O_2_ and O_2_^·−^ accumulation in leaves of *ThASR3*-transformed and WT *Arabidopsis* plantlets. **C**-**F** Analysis of H_2_O_2_ (**C**) and MDA (**D**) contents, SOD (**E**) and POD (**F**) activities in four-week-old *ThASR3*-transformed transgenic and WT *Arabidopsis* plantlets under 200 mM NaCl or 300 mM mannitol treatment condition. Plants were selected for photography in each treatment, and the most representative photos were selected and combined. The asterisks (*) indicate a significant difference (*P* < 0.05) between transformed (OE and IE) and control (VC) plants

The accumulation of H_2_O_2_ and O_2_·^−^ in VC, OE, and IE *T. hispida* plantlets was investigated under salt and osmotic stresses using DAB and NBT staining. The results showed that the staining intensity remarkably decreased in OE plants branches but increased in IE plants, compared with VC plants under stress conditions, suggesting that *ThASR3* positively decreases accumulation of H_2_O_2_ and O_2_·^−^ (Fig. 5A, B). Moreover, H_2_O_2_ and MDA contents exhibited significantly lower in OE lines under salt and mannitol treatments while observably higher in RNAi plants, compared to VC plants (Fig. 5C, D). To validate the results in *T. hispida*, we detected ROS accumulation and H_2_O_2_ or MDA contents in transgenic *Arabidopsis* plants overexpressing *ThASR3*, which is consistent with the result in *T. hispida* (Fig. 6C, D).

In addition, we further analyze the activities of the SOD and POD to demonstrate whether they contribute to the reduction of ROS. Under stress conditions, the SOD and POD activities were significantly increased in OE plants and reduced in IE plants, compared with VC plants (Fig. 5E, F). Consistently, the activities of POD and SOD in *ThASR3*-transformed *Arabidopsis* plants were significantly increased compared with WT plants under abiotic stress conditions (Fig. 6E, F). These results suggest that *ThASR3* decreased ROS accumulation by reducing H_2_O_2_ content and enhancing POD and SOD activities in transgenic *T. hispida* and *Arabidopsis*.

### *ThASR3* can reduce cell membrane damage

**Fig. 7:**
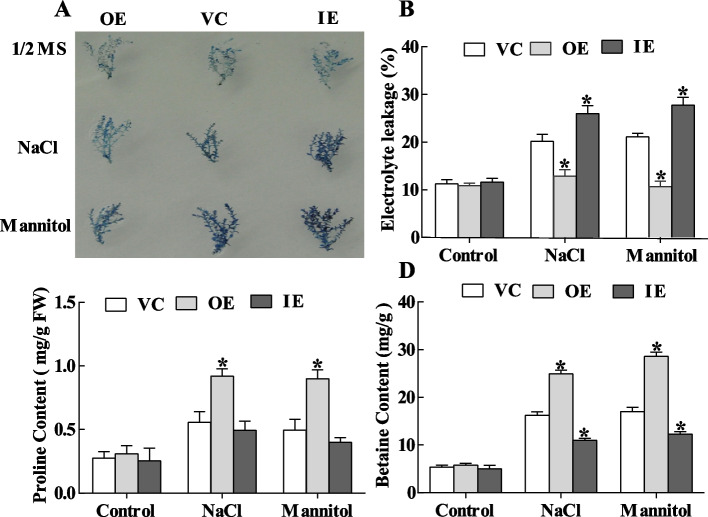
Detection of cell death in three types (VC, OE and IE) transgenic *T. hispida* plantlets. **A** Evans blue staining. Fresh young branches from *ThASR3*-transformed *T. hispida* plantlets were harvested to detect Evans blue staining under 200 mM NaCl or 300 mM mannitol treatment. **B**-**D** Electrolyte leakage **B**, proline **C**, and betaine contents **D** in three types (VC, OE and IE) transgenic *T. hispida* plantlets grown for 24 hours on 1/2 MS solid medium supplemented with 100 mM NaCl or 200 mM mannitol. 15 plants were selected for photography in each treatment, and the most representative photos were selected and combined. The asterisks (*) indicate a significant difference (*P* < 0.05) between transformed (OE and IE) and control (VC) plants

**Fig. 8:**
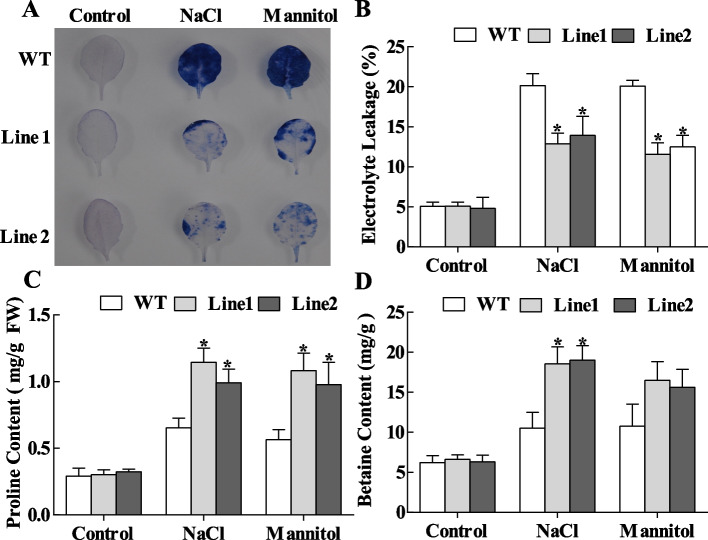
Detection of cell death in *ThASR3-*overexpressing *Arabidopsis* and WT plants. **A** Evans blue staining. Young leaves from *ThASR3*-transformed, and WT *Arabidopsis* plantlets were obtained to detect Evans blue staining under 200 mM NaCl or 300 mM mannitol treatment conditions. **B**-**D** Electrolyte leakage **B**, proline **C**, and betaine contents **D** in transgenic and WT *Arabidopsis* plants. 15 plants were selected for photography in each treatment, and the most representative photos were selected and combined. The asterisks (*) indicate a significant difference (*P* < 0.05) between transformed (OE and IE) and control (VC) plants

We further analyzed cell membrane damage using Evans blue staining, with the intensity of the staining representing the degree of cell membrane damage. Evans blue staining was not substantially different among the three types of *T. hispida* plants (VC, OE, IE) under normal conditions. However, the intensity was significantly reduced in OE plants but increased in RNAi plants under salt and mannitol conditions, compared to VC plants (Fig. 7A). Meanwhile, we found that the area of Evans blue staining in transgenic *Arabidopsis* leaves was significantly smaller than that in WT plants under stress conditions (Fig. 8A)*.* Furthermore, we further measured the electrolytic leakage rate in transgenic *T. hispida* plants. The results showed that the electrolytic leakage rate had no difference under normal conditions among VC, OE, and IE plants. However, it was significantly reduced in OE plants but increased in IE plants under salt and mannitol stress conditions, compared to VC plants (Fig. 7B). Similar results were obtained in transgenic *Arabidopsis* plants, compared to WT plants under stress conditions (Fig. 8B). Collectively, these results suggest that overexpression of *ThASR3* markedly mitigates cell membrane damage in transgenic *T. hispida* and *Arabidopsis*.

### *ThASR3* can increase the contents of proline and betaine

Proline and betaine contents served as osmotic adjustments to protect plant cells from abiotic stresses. Once stress is eased, the accumulated proline might be dissolved as an energy supply for plant development [36]. In our study, proline and betaine contents were positively increased in OE plants and reduced in IE plants, compared to VC plants under salt and osmotic stresses (Fig. 7C, D). Similarly, overexpressing-*ThASR3 Arabidopsis* plants dramatically enhanced proline and betaine contents under salt and osmotic stresses conditions (Fig. 8C, D). These results suggest that overexpression of *ThASR3* increases proline and betaine biosynthesis, further contributing to the osmotic potential, eventually improving abiotic stress tolerance.

## Discussion

*Tamarix hispida*, a woody halophyte, is highly tolerant to salinity and drought, which indicates that there are some efficient abiotic stress tolerance genes in *Tamarix hispida*. Previous studies have reported that the *ASR* gene family is involved in response to multiple abiotic stresses and molecular signaling pathways [13, 17, 27, 37]. However, the functional elucidation of *ASRs* is still unclear in *T. hispida*. In the present study, we identified and characterized the function of an *ASR* gene in *T. hispida*. We provide evidence that *ThASR3* functions as a positive regulator in *Tamarix* responses to salt and osmotic stresses by enhancing ROS scavenging and accumulation of osmoprotectant.

Multiple sequence alignment results show that ThASR3 contained the main conserved ABA/WDS domain, histidine-rich and alanine-rich area similar to ASR family (Fig. 1A), which also exists in various species, such as wheat, maize, rice and poaceae [16, 26, 37, 38]. The ASR protein contains two highly conserved regions, including a nuclear localization signal region (rich in lysine-based) near the C-terminus and a histidine-rich region near the N-terminus [39]. The N-terminal consensus sequence of most ASRs contains six His residues. For halophytes, the N-terminal is rich in glycine, and myristoylation mostly occurs on the N-terminal glycine. The myristoylation at the N-terminal is associated with signaling pathways during salt stress adaptation. ThASR3 contains two glycine residues at the N-terminus, which is consistent with the structural characteristics of halophyte ASRs [40, 41].

Recently, many *ASR* family members have been found to be involved in response to various environmental stresses. For example, the expression of *TtASR* was induced by salt, osmotic stress, and ABA treatments in *Tetragonia tetragonoides* [42]. Over-expression of *BdASR4* increases drought tolerance of transgenic *Brachypodium distachyon L* [25]. Over-expression of *OsASR1* and *OsASR3* can increase the tolerance of salt and drought stresses in transgenic rice [43]. In our study, *ThASR3* was proved to be induced by salt and drought stresses (Fig. 2A, B). And transgenic *Arabidopsis* overexpressing *ThASR3* showed significantly growth advantage under stress conditions (Fig. 3A, D). The OE plants with the significant highest expression, and the IE plants with the significant lowest expression were selected for the gain- and loss-of-function characterization of *ThASRs* in transgenic *Tamarix*. *ThASR3* was significantly reduced in IE plants but increased in OE plants under stress conditions, compared to VC plants (Fig. 4). These results suggest that *ThASR3* functions as a stress-responsive gene and enhances salt and drought tolerance in transgenic *Tamarix*. These results are consistent with previous studies on *ASR* genes from other plant species [20, 29, 44].

Adverse environments, including salt, drought, heat, and cold, can cause rapid accumulation of ROS in plants and then induce multiple degrees of cell membrane damage through the oxidation of proteins, lipids and DNA [45, 46]. Therefore, the scavenging capability of ROS plays a crucial role in protecting plants against oxidative stress. Two common ROS species including H_2_O_2_ and O_2_·^−^ are vital signaling molecules in plant cells. Overexpression of maize *ZmASR3* decreases H_2_O_2_ accumulation in transgenic *Arabidopsis* [47]. TaASR1-D confers salt and osmotic stress resistance by affecting ROS accumulation in transgenic wheat [48]. In this study, NBT and DAB histochemical staining showed that ROS accumulation in transgenic *Tamarix* and *Arabidopsis* plants overexpressing-*ThASR3* was remarkably reduced compared with control (VC or WT) plants under salt and osmotic stresses (Figs. 5A, B and 6A, B). Consistently, SOD and POD enzyme activities were lower in *ThASR3* RNAi plants, and higher in *ThASR3* OE plants (Figs. 5E, F and 6E, F), compared to VC plants. In addition, our results showed that the H_2_O_2_ and MDA contents, and electrolyte leakage were significantly decreased in OE plants under salt and osmotic treatments (Figs. 5C, D, 6C, D, 7B and 8B). Collectively, our study provided the physiological evidence that *ThASR3* confers salt and osmotic stress tolerance by improving the antioxidant system and minimizing lipid peroxidation to enhance ROS scavenging capability in vivo.

Compatible solutes such as proline and betaine play important roles in plant stress tolerance. Plant proline functions as a free radical scavenger and osmotic agent, protecting cells from harm and sustaining long-term growth under abiotic stresses conditions [49]. In this study, overexpressed-*ThASR3* transgenic *Arabidopsis* showed higher proline and betaine contents than WT plants (Fig. 8C, D). And transgenic *Tamarix* plants overexpressing-*ThASR3* also displayed increased proline and betaine contents under salt and osmotic stresses, compared to IE and VC (Fig. 7C, D). The results demonstrate that *ThASR3* promotes the biosynthesis of proline and betaine in plants, contributing to osmotic potential to improve stress tolerance.

To date, *Tamarix* has no stable transformation system. In this study, transgenic *Tamarix* plants were obtained by a transient transformation method. However, it is impossible to compare the phenotype of transient transgenic *Tamarix* plants. To compensate for this deficiency, we performed phenotypic analysis using *Arabidopsis* with ectopic expressing *ThASR*. It is well known that homologous expression systems are more precise than heterologous expression in functional characterization of plant genes. Therefore, at present, we are constructing a genetic transformation system suitable for *Tamarix* plants, which will provide a tool to validate the results of this study using *Tamarix* homologous expression system.

## Conclusions

In this study, a salt and drought-induced *ASR* gene, *ThASR3,* was cloned from *T. hispida* and functionally characterized. Transgenic *Arabidopsis* overexpressing *ThASR3* displayed growth and physiological advantages compared with wild-type plants under both salt and drought stresses. Overexpression of *ThASR3* in transgenic *Tamarix* also confers high salt and osmotic stress tolerance, which was reflected from higher.

SOD and POD activities, proline and betaine contents and lower H_2_O_2_ content, electrolyte leakage and malondialdehyde, compared to *ThASR3* RNAi-silencing and control plants. Moreover, the DAB, NBT and Evans blue intensity was significantly reduced in OE plants but increased in RNAi plants under salt and mannitol conditions, compared to VC plants. All the results indicated *ThASR3* improves salt and osmotic tolerances of transgenic plants by enhancing ROS-scavenging capability and osmotic adjustment ability. This study improves our understanding of the positive functions of *ThASR3* in salinity and osmotic tolerance in *T. hispida* and proves theoretical foundation for characterization of *ASR* genes in woody plants.

## Materials and methods

### Plant materials

*T.hispida* plantlets were cultivated in peat and sand mixture (2:1 v/v) in the culture room with conditions of light/dark cycles of 14 h/10 h, 70–75% relative humidity, and an controlled average temperature of 24 °C. Four-week-old plantlets were irrigated with water (as control), 400 mM NaCl and 20% (w/v) PEG6000, respectively. After 1, 2, 6, 12, 24 and 48 h, the tissue samples were harvested for gene expression analysis. At least 15 seedlings were pooled in each sample, all stress treatment experiments were repeated three times, each with three technical replicates. At the same time, a fresh water-only control was conducted in parallel. *Arabidopsis* seeds (ecotype Columbia) were sterilized in 5% (v/v) sodium hypochlorite before germinated on half-strength Murashige and Skoog (1/2 MS) solid medium plates. *Arabidopsis* plants were cultivated in the mixture of soil, vermiculite and perlite mixture (5:3:2 v/v) in the culture room with 70–75% relative humidity at a constant temperature of 22 °C and a light/dark photocycle of 16 h/8 h.

### Cloning of *ThASR3* and sequence analysis

The full-length transcript sequence of *ThASR3* (Genbank accession number: OL310472) was cloned based on the transcriptome of *T. hispida* [34]. ThASR3 and other ASRs amino acid sequences from different plant species were aligned using Bioedit software. The phylogenetic tree was built via neighbor-joining method. Conserved domains were analyzed using NCBI Conserved Domain Database (https://www.ncbi.nlm.nih.gov/Structure/cdd/wrpsb.cgi).

### qRT-PCR analysis

CTAB (hexadecyltrimethylammonium bromide) method was performed to isolate total RNA from *T. hispida* plants. Briefly, the sample (100 mg) powdered in liquid nitrogen, was added to the extraction buffer (2% CTAB, 2.5% PVP-40, 2 M NaCl,100 mM Tris-HCl pH 8.0, 25 mM EDTA pH 8.0 and 2% of β-mercaptoethanol) at 65 °C for 10 min. An equal volume of chloroform:isoamyl alcohol (24:1 v/v) was added. LiCl (3 M final concentration) was added and resuspended in SSTE buffer (10 mM Tris-HCl pH 8.0, 1 mM EDTA pH 8.0, 1% SDS, 1 M NaCl), an equal volume of chloroform:isoamyl alcohol was added. The RNA was precipitated with 0.7 vols of cold isopropanol and washed with ethanol (70%), dried and resuspended in DEPC-water [50, 51]. The PrimeScript™ RT Reagent Kit was used to synthesize first-strand cDNA (TaKaRa, China). Real-time qRT-PCR was carried out following the protocol described by Wang [52]. *ThAlpha tubulin*, *ThBeta tubulin and ThActin* genes were used as internal reference genes (Supplementary Table S2) [10]. The efficiency of all primers used for qRT-PCR was close to 1 and reference genes were approximately equal. The 2^−ΔΔCT^ method was used to detect the relative expression levels of genes [53]. The relative mRNA expression was calculated as the transcription level under stress treatment divided by the transcription level under control conditions (the samples without treatment, were harvested at the corresponding time points). The relative expression level was log_2_ transformed. In this way, the value (scale) > 0 mean up-regulate, =0 mean unregulated, and < 0 means down-regulated. All the primer sequences were list in Table S2. For each sample, at least three biological replicates and three technical replicates were conducted.

### Vector construction and generation of *ThASR3* transformed plants

The full-length coding sequence (CDS) of *ThASR3* was fused into plant binary expression vector pROKII under the control of CaMV 35S promoter (35S::*ThASR3*) to generate overexpression construction. The pROKII was double digested by *smaI* and then were ligated by Infusion ligase. The recombinant plasmid pROKII*-ThASR3* was detected by PCR using specific vector primers (Supplementary Table S1). An inverted repeat truncated cDNA of *ThASR3* was inserted into the RNAi vector pFGC5941 on both sides of CHSA intron, which was used to silence *ThASR3* [13]. The amplified fragment and the plant binary expression vector pFGC5941 were double digested by *BamH* and *XbaΙ* and then were ligated together by T4 ligase (Promega, China). The recombinant plasmid *ThASR3*-pFGC5941 was detected by PCR using specific vector primers (Supplementary Table S1). All the primers used were exhibited in Table S1. The 35S::*ThASR3* and pFGC5941-*ThASR3* were transferred into *Agrobacterium tumefaciens* strain EHA105 by freeze-thaw method. Transient transformation of 6-week-old entire seedlings was carried out based on Ji’s approach with certain changes [54]. Briefly, the whole plant seedlings were soaked in the 1/2 MS transformation solution [150 mM acetosyringone, 2.5% (w/v) sucrose, 0.01% (w/v) Tween-20, pH 5.8] with *Agrobacterium tumefaciens* EHA105 strain at 0.6 OD_600_ and incubated with shaking at 120 rpm for 4 h at 25 °C. Then, the seedlings were washed twice with distilled water and gently wiped with sterile paper. The plantlets were grown vertically on 1/2 MS agar medium [150 mM acetosyringone, 2.5% (w/v) sucrose, pH 5.8] in tissue culture bottles. The floral dip transformation method was performed to generate transgenic *Arabidopsis* lines [55]. Briefly, the centrifuged cells were adjusted to an OD_600_ of 0.8 with the transformation solution [150 μM acetosyringone, 5% (w/v) sucrose, 0.02% (w/v) Silwet-77 and 100 μM Triton X-100]. Seeds from T_0_ transgenic plants were plated in kanamycin selection medium (50 mg·L^− 1^). The positive transgenic lines were selected on kanamycin (50 mg/L) plates, and further identified by genomic DNA PCR, and the *ThASR3* expression level of each transgenic line was examined by qRT-PCR. The homozygous lines of T_3_ generation plants were used for study. For each sample, at least three biological replicates and three technical replicates were conducted.

### Stress tolerance analysis of transgenic lines

The seeds of two homozygous *ThASR3*-overexpressing transgenic *Arabidopsis* and wild-type (WT) plants were sterilized and grown on 1/2 MS medium for 10 days. Then they were transferred to 1/2 MS medium with 100 mM NaCl or 200 mM mannitol at 22 °C for 7 days, respectively. The root growth and fresh weight of transgenic *Arabidopsis* and WT seedlings were examined. For salt and osmotic tolerance test in soil, one-month-old seedlings were watered with 200 mM NaCl and 300 mM Mannitol for 7 days with continued watering as control. A minimum of three biological replicates, which contain at least 45 transgenic plants in each replicate, were performed to ensure the accuracy of each stress tolerance assay. The most representative individuals were used for photograph.

### DAB and NBT staining

3,3′- diaminobenzidine (DAB) and nitroblue tetrazolium (NBT) staining were performed to detect H_2_O_2_ and superoxide (O_2_^·−^). The transformed *T. hispida* plantlets were exposed to 100 mM NaCl or 200 mM mannitol treatment for 24 h, and one-month-old transformed *Arabidopsis*and WT seedlings were treated with 200 mM NaCl or 300 mM mannitol for 2 h, respectively. Approximately 20 branches harvested from *T. hispida* and 20 young leaves obtained from *Arabidopsis* were respectively incubated with Evans blue (10 mg/mL), NBT (10 mg/mL) or DAB (10 mg/mL) solutions according to the descriptions by Zang [10]. At least three biological replicates were conducted for each experiment,each with three technical replicates.

### Physiological changes involve in abiotic stress tolerance

The superoxide dismutase (SOD) and peroxidase (POD) activities, and malondialdehyde (MDA), H_2_O_2_ and proline contents were detected using Nanjing Jiancheng Bioengineering Institute reagent kits (China) as directed by the manufacturer. The catalog numbers of these reagent kits are as follows: A064–1 (H_2_O_2_), A001–1 (SOD), A084–3 (POD), A003–1(MDA), and A107–1-1(proline). The betaine content was measured by JiangSu Kemin Institute reagent kit (TCJ-2-G, China) as directed by the manufacturer. Electrolyte leakage was performed according to the methods of Ben-Amor [56]. Three biological repeats were performed and at least 15 seedlings were used for per sample, each with three technical replicates.

### Statistical analyses

SPSS19 software was used for statistical analyses, completely randomized design was used, and the statistically significant (*, *P* < 0.05) were considered as significant differences.

## Supplementary Information

**Additional file 1.** Supplemental data.

## Data Availability

The datasets generated and /or analyzed during the current study are available in NCBI with the accession number OL310472. The direct link for the NCBI database is https://www.ncbi.nlm.nih.gov/search/all/?term=OL310472.

## Abbreviations

ASR: abscisic acid-, stress-, and ripening-induced
ROS: reactive oxygen species
MS: Murashige and Skoog medium
PCR: polymerase chain reaction
SOD: superoxide dismutase
POD: peroxidase
MDA: malondialdehyde
NBT: nitroblue tetrazolium
DAB: 3,3′-Diaminobenzidine
