# Supplementary material for: ThASR3 confers salt and osmotic stress tolerances in transgenic Tamarix and Arabidopsis
Source: BMC Plant Biol. 2022 Dec 14;22:586. doi: 10.1186/s12870-022-03942-w (PMC9749169; doi:10.1186/s12870-022-03942-w)
Supplement: Supplementary file 1 — Additional file 1. Supplemental data. [file 12870_2022_3942_MOESM1_ESM.doc]

**Supplement Data**


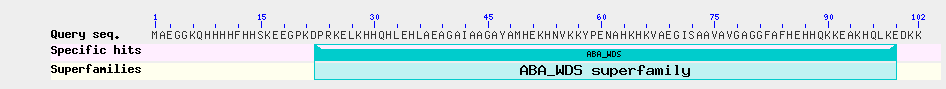


**Fig. S1** The domain of ThASR3 protein


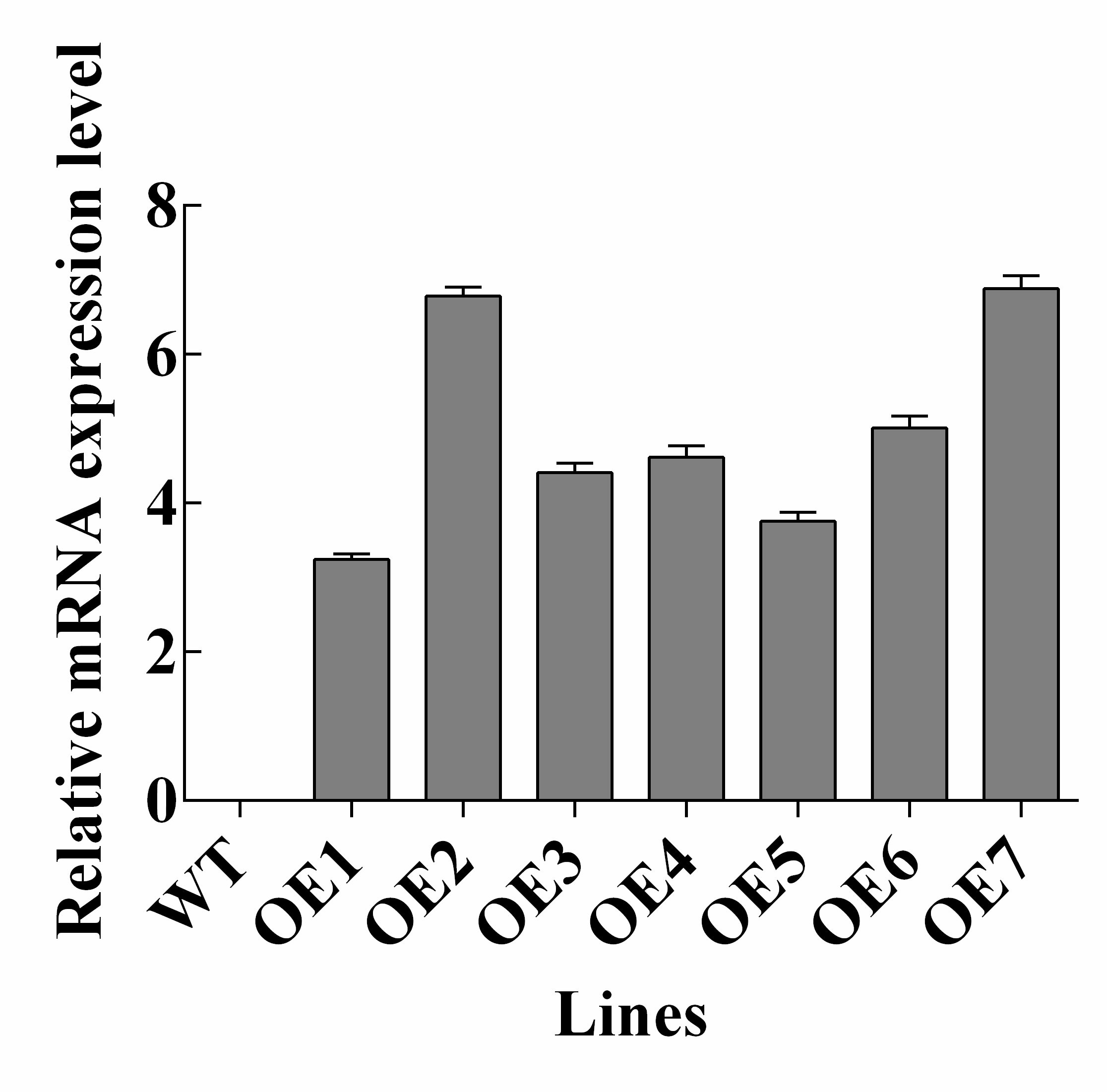


**Fig. S2** qRT-PCR detection of *ThASR3* mRNA expression level of transgenic *Arabidopsis*

**Supplementary Table S1. Primer sequences used in construction of overexpressing *ThASR3*, RNAi-silenced *ThASR3* vectors.**

| **Primer names** | **Forward primers (5’- 3’)** | **Reverse primers (3’- 5’)** |
| --- | --- | --- |
| *ThASR3*-prokII | CTCTAGAGGATCCCCATGGTTGAAGGAGGAAAGC | TCGAGCTCGGTACCC TTACTTTATGTCTTCTTTGAG |
| pROKII | CTCCACTGACGTAAGGGAT | CGCAAGACCGGCAACAGGAT |
| *ASR3*-Cis | ATTACCATGGGGCGCGCC ATGGCTGAAGGAGGAAAGC | TCATCGATTGGGCGCGCC TTACTTTTTGTCTTCTTTGAG |
| *ASR3*-Anti | CCTAGGTGAGTCTAGAGCCAGAAATGTTTGCACCAACTG | TAATTAACTCTCTAGACTGATGCTAGACACCCGGATGC |
| pFGC-Anti | CTTACTTACACTTGCCTTGGAG | ATCTGAGCTACACATGCTCAG |
| pFGC-Cis | ATAAGGAAGTTCATTTCATTTG | CAATCAAATGAAGAGCCAAT |

Supplementary Table S2. The primer sequences used in real time RT-PCR.

| **Gene name** | **Forward primers (5'-3')** | **Reverse primers (5'-3')** |
| --- | --- | --- |
| *ThASR3* | GCTGAAGGAGGAAAGCAAC | CTTTTTGTCTTCTTTGAGTTG |
| *ThAlpha tubulin* | CACCCACCGTTGTTCCAG | ACCGTCGTCATCTTCACC |
| *ThBeta tubulin* | GGAAGCCATAGAAAGACC | CAACAAATGTGGGATGCT |
| *ThActin* | AAACAATGGCTGATGCTG | ACAATACCGTGCTCAATAGG |
